# Supplementary material for: A survival selection strategy for engineering synthetic binding proteins that specifically recognize post-translationally phosphorylated proteins
Source: Nat Commun. 2019 Apr 23;10:1830. doi: 10.1038/s41467-019-09854-y (PMC6478843; doi:10.1038/s41467-019-09854-y)
Supplement: Supplementary file 1 — Supplementary Information [file 41467_2019_9854_MOESM1_ESM.pdf]

Supplementary Information

A survival selection strategy for engineering synthetic binding proteins that specifically recognize post-translationally phosphorylated proteins

Bunyarit Meksiriporn, Morgan B. Ludwicki, Erin A. Stephens, Allen Jiang, Hyeon-Cheol Lee, Dujduan Waraho-Zhmayev, Lutz Kummer, Fabian Brandl, Andreas Plückthun, and Matthew P. DeLisa

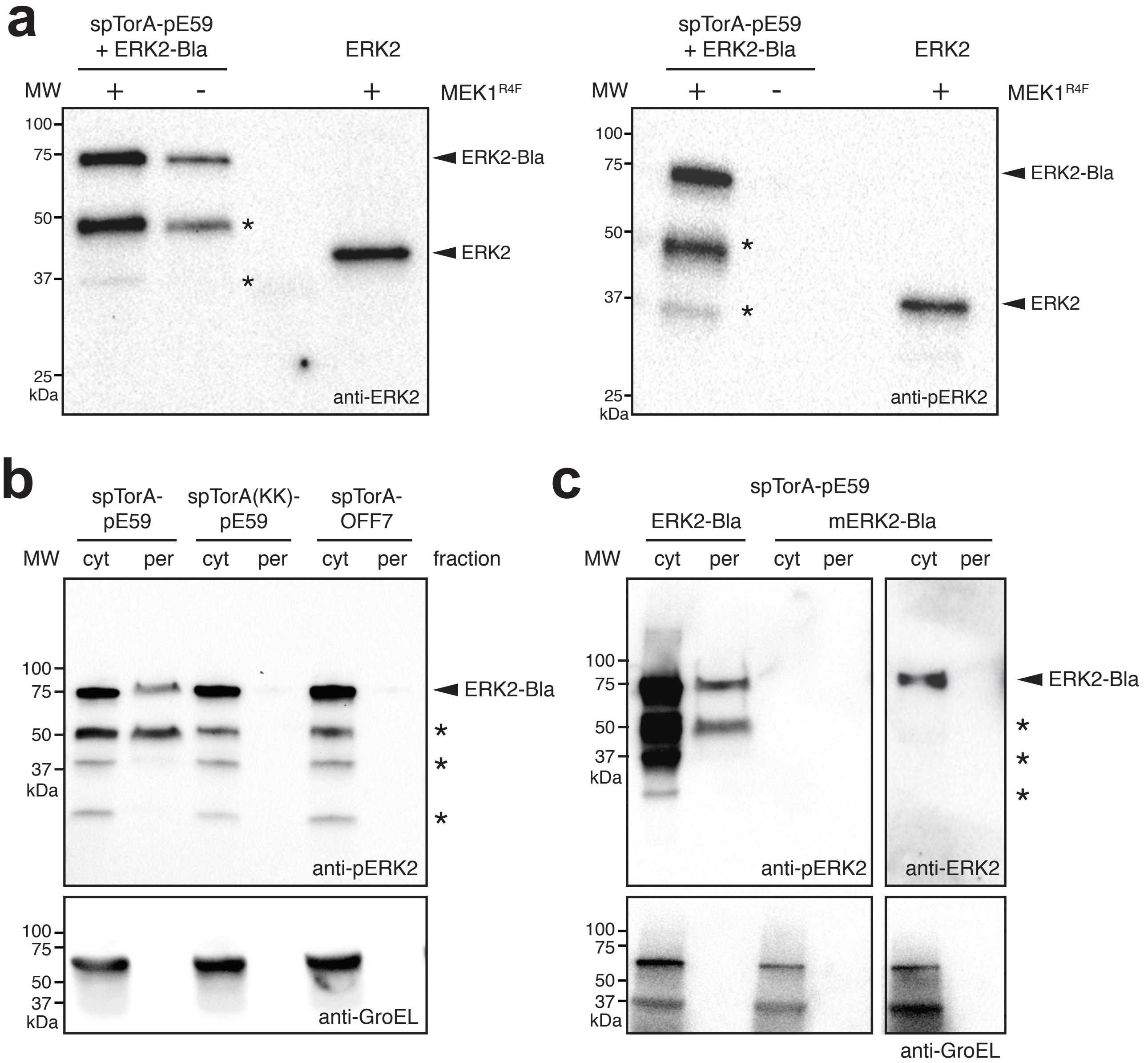

Supplementary Figure 1. Supplementary Figure 1. Expression and subcellular localization of PhLI-TRAP proteins. (a) Western blot analysis of cytoplasmic fractions derived from MC4100 cells co-expressing TatABC along with the antigen-Bla reporter fusion (ERK2-Bla) and Tat-targeted DARPin (spTorA-pE59) in the presence (+) or absence (-) of kinase MEK1^{R4F} (lanes 1 and 2). The cytoplasmic fraction from MC4100 cells expressing unfused ERK2 from plasmid pET-His6-ERK2-MEK1_R4F_coexpression (Addgene # 39212) was included as a positive control (lane 4). Lane 3 was empty. Duplicate membranes were prepared by SDS-PAGE followed by blotting of separated proteins onto PVDF membranes. One membrane was probed with anti-ERK2 antibody (left) and the other with anti-pERK2 antibody (right) to detect the non-phosphorylated and phosphorylated ERK2 forms, respectively. (b) Western blot analysis of cytoplasmic (cyt) and periplasmic (per) fractions prepared from MC4100 cells co-expressing TatABC along with the antigen-Bla reporter fusion (ERK2-Bla) and a DARPin (spTorA-pE59, spTorA(KK)-pE59, or spTorA-OFF7) in the presence (+) or absence (-) of kinase MEK1^{R4F}. Membrane was probed with anti-pERK2 antibody. Quality of fractions was confirmed by probing membrane with an anti-GroEL antibody. (c) Same as in (b) but with fractions prepared from MC4100 cells co-expressing TatABC and spTorA-pE59 along with an antigen-Bla reporter fusion (ERK2-Bla or mERK2-Bla) in the presence (+) or absence (-) of kinase MEK1^{R4F}. Membranes were probed with anti-pERK2 antibody (left), anti-pERK2 antibody (right), and anti-GroEL antibody (bottom). Asterisks denote degradation products. Molecular weight (MW) markers are indicated at left. All results are representative of at least three biological replicates.

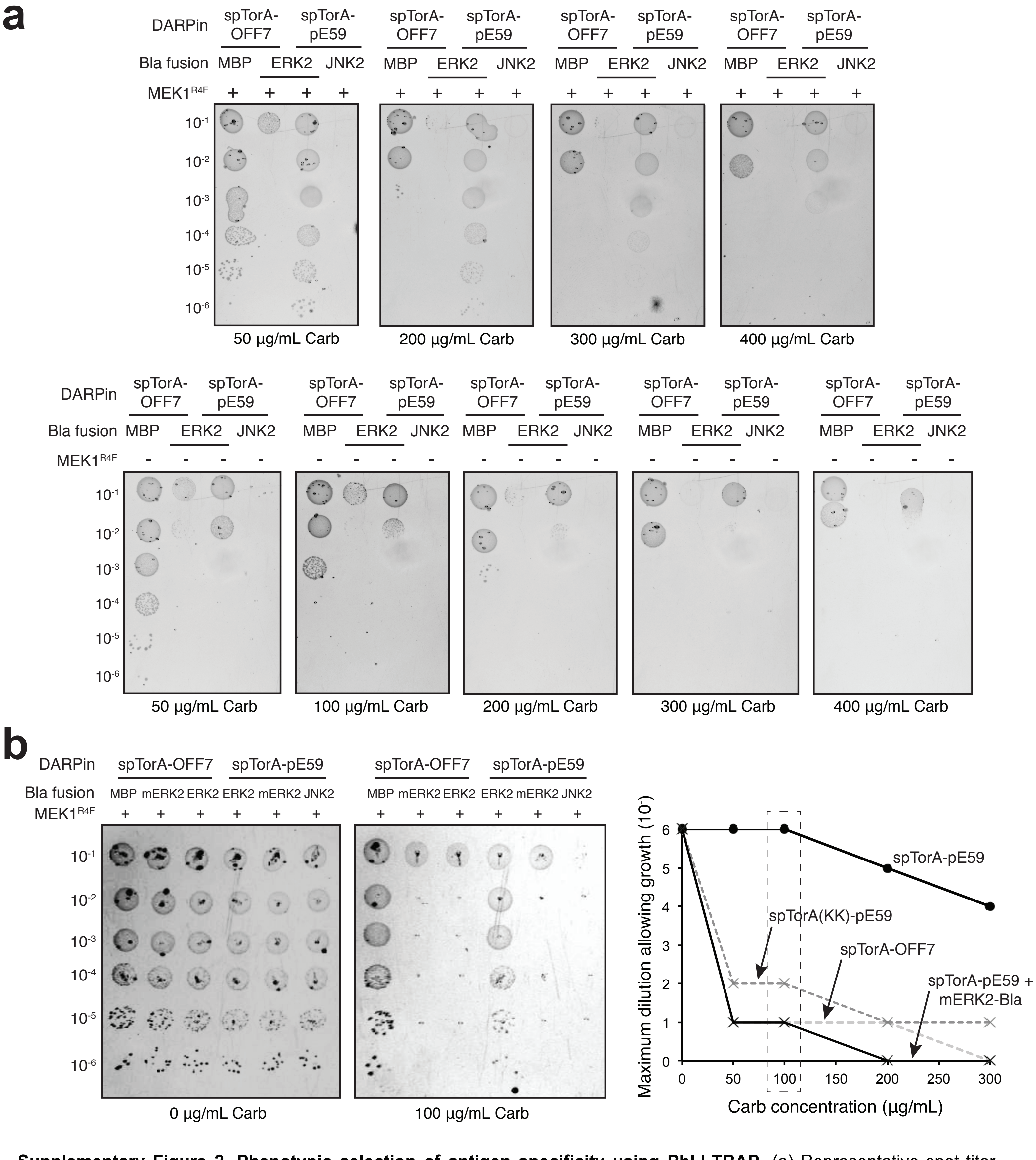

Supplementary Figure 2. Phenotypic selection of antigen specificity using PhLI-TRAP. (a) Representative spot titer images for serially diluted *E. coli* MC4100 cells co-expressing TatABC along with the antigen-Bla reporter fusion (MBP-Bla, ERK2-Bla, or JNK2-Bla) and a Tat-targeted DARPin (spTorA-pE59 or spTorA-OFF7) as indicated. Resistance of cells was evaluated in the presence (+) or absence (-) of MEK1^{R4F} kinase. Overnight cultures were serially diluted in liquid LB and plated on LB agar supplemented with Carb (50-400 µg/ml). (b) Representative spot titer (left) and survival curves (right) for serially diluted *E. coli* MC4100 cells co-expressing TatABC along with the antigen-Bla reporter fusion (MBP-Bla, mERK2-Bla, ERK2-Bla, or JNK2-Bla) and a Tat-targeted DARPin (spTorA-pE59 or spTorA-OFF7) as indicated. Resistance of cells was evaluated in the presence (+) or absence (-) of MEK1^{R4F}. Maximal cell dilution that allowed growth is plotted versus Carb concentration. Dashed box indicates Carb concentration that is depicted in image panel and corresponds to 100 µg/ml Carb.

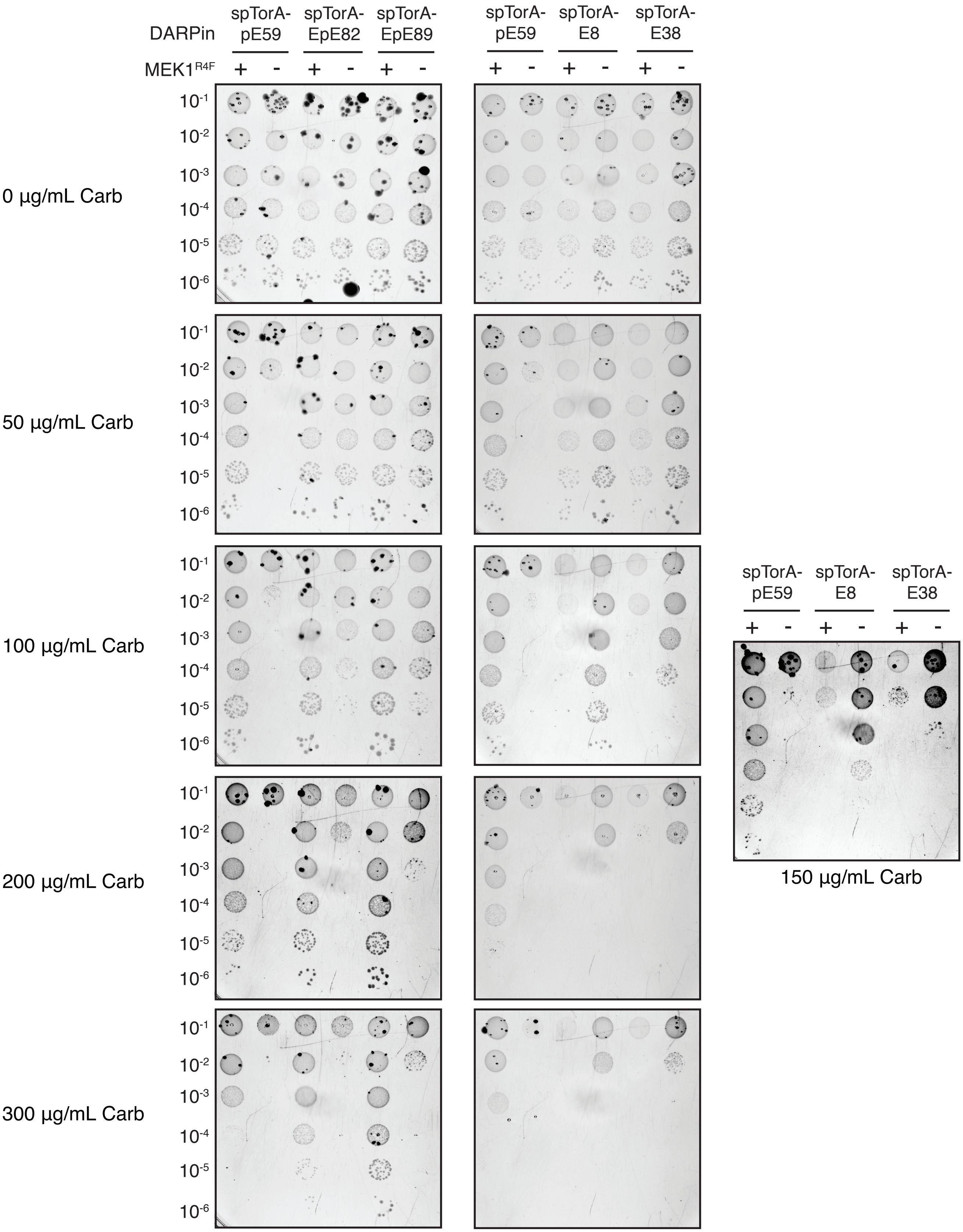

Supplementary Figure 3. Phenotypic selection of antigen selectivity using PhLI-TRAP. Representative spot titer images for serially diluted *E. coli* MC4100 cells co-expressing TatABC along with the antigen-Bla reporter fusion (ERK2-Bla) and a Tat-targeted DARPin (spTorA-pE59, spTorA-EpE82, spTorA-EpE89, spTorA-E8, or spTorA-E38) as indicated. Resistance of cells was evaluated in the presence (+) or absence (-) of MEK1^{R4F} kinase. Overnight cultures were serially diluted in liquid LB and plated on LB agar supplemented with Carb (50-300 µg/ml). Maximal cell dilution that allowed growth is plotted versus Carb concentration.

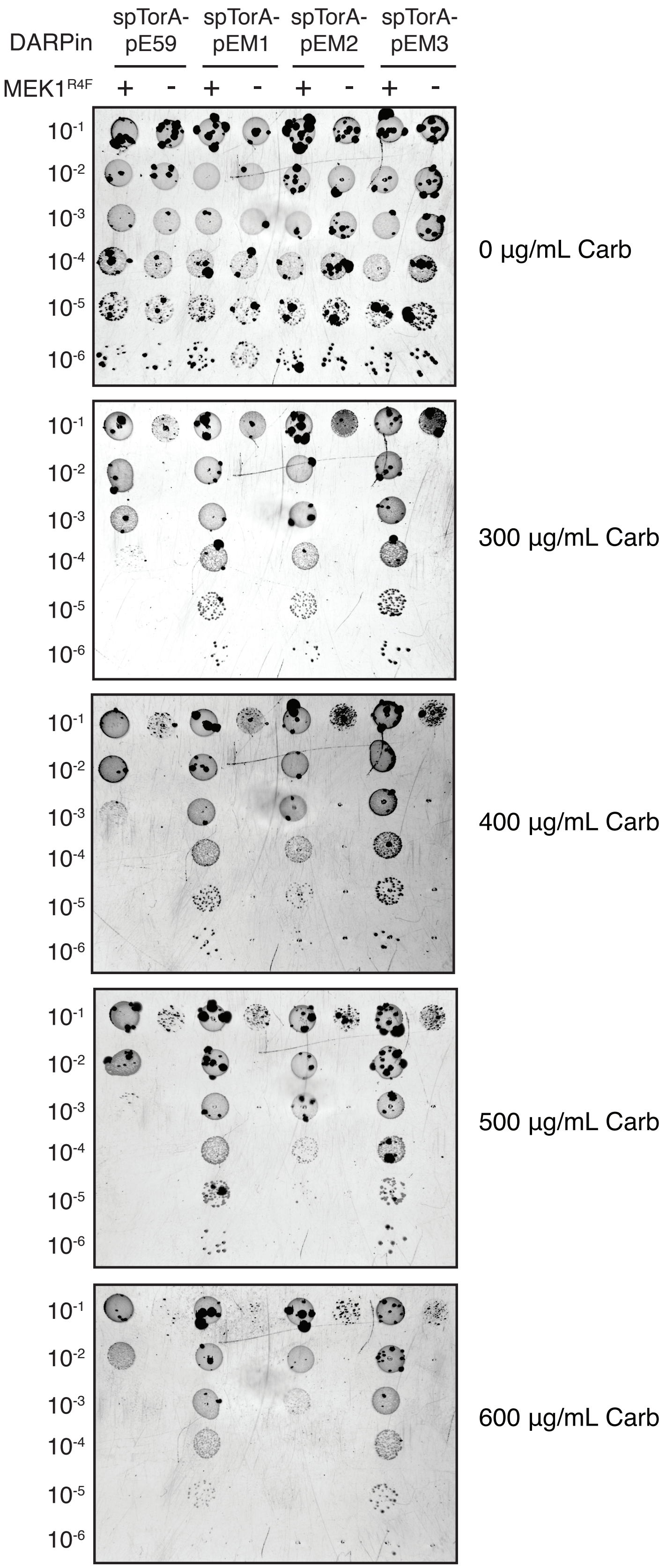

Supplementary Figure 4. Phenotypic selection of affinity matured DARPin variants isolated using PhLI-TRAP. Representative spot titer images for serially diluted *E. coli* MC4100 cells co-expressing TatABC along with the antigen-Bla reporter fusion (ERK2-Bla) and a Tat-targeted DARPin (spTorA-pE59, spTorA-pEM1, spTorA-pEM2, or spTorA-pEM3) as indicated. Resistance of cells was evaluated in the presence (+) or absence (-) of MEK1^{R4F} kinase. Overnight cultures were serially diluted in liquid LB and plated on LB agar supplemented with Carb (0-600 µg/ml).

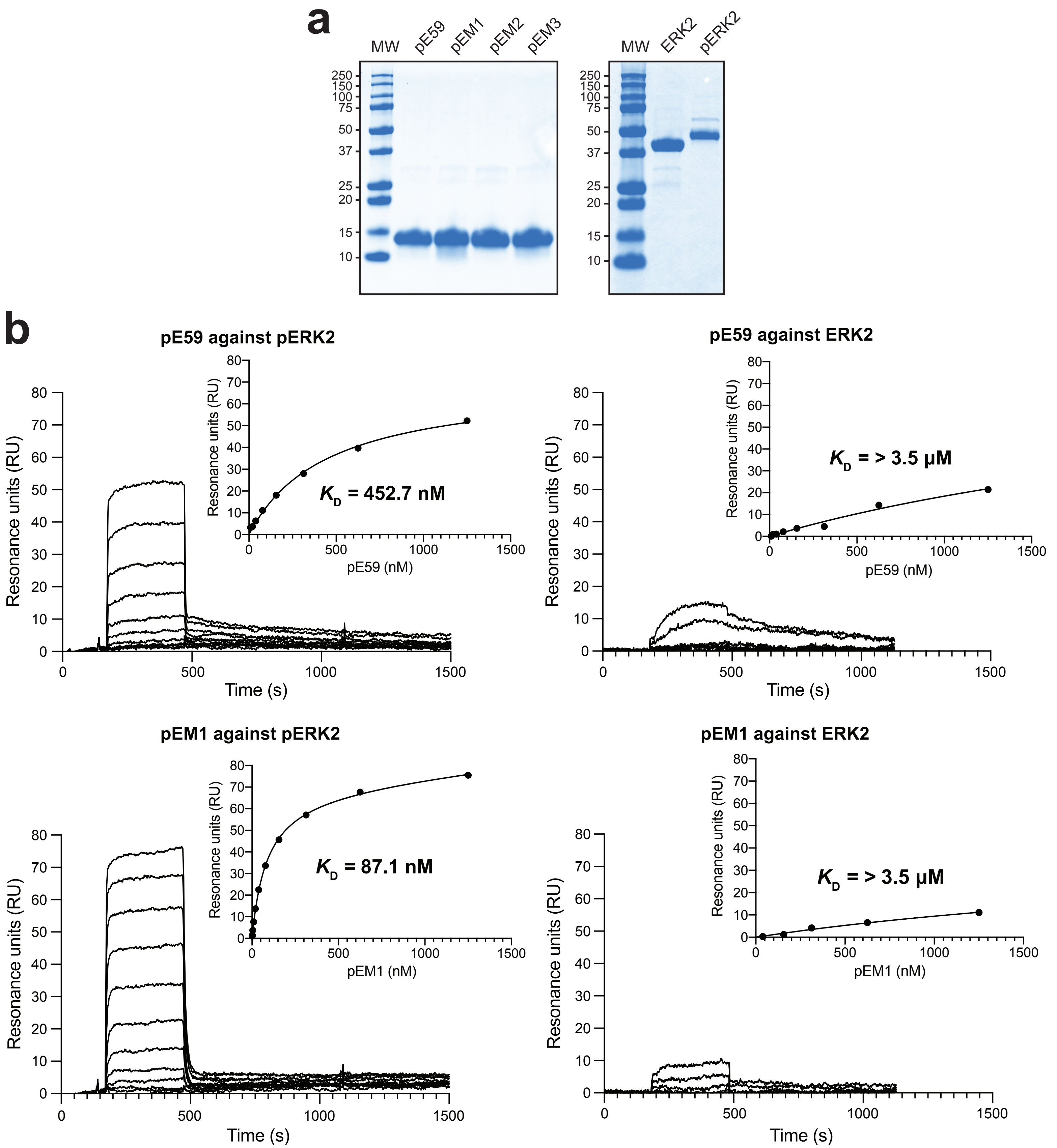

Supplementary Figure 5. Affinity determination of DARPin variants pEM1, pEM2, and pEM3. (a) Representative coomassie-stained SDS-PAGE gels loaded with the different DARPins (left panel) and the biotinylated ERK2 and pERK2 antigens (right panel) following expression and purification. Purity of all proteins was estimated to be >95%. Molecular weight (MW) markers are loaded in lane 1 of each gel. (b) The binding kinetics of variants pEM1, pEM2, and pEM3 to the ERK2 and pERK2 antigens were monitored using Biacore. Biotinylated ERK2 and pERK2 were immobilized at low concentrations, and the response of varied amounts of each DARPin (0, 1.2, 2.4, 4.9, 9.8, 19.5, 39, 78.125, 156.25, 312.5, 625, 1250 nM) was compared with an empty flow cell. Three independent experiments were carried out for each combination. Representative sensorgram results are depicted. The data were evaluated by fitting the equilibrium binding responses to obtain affinity values with resulting curve fits depicted next to the sensorgrams.

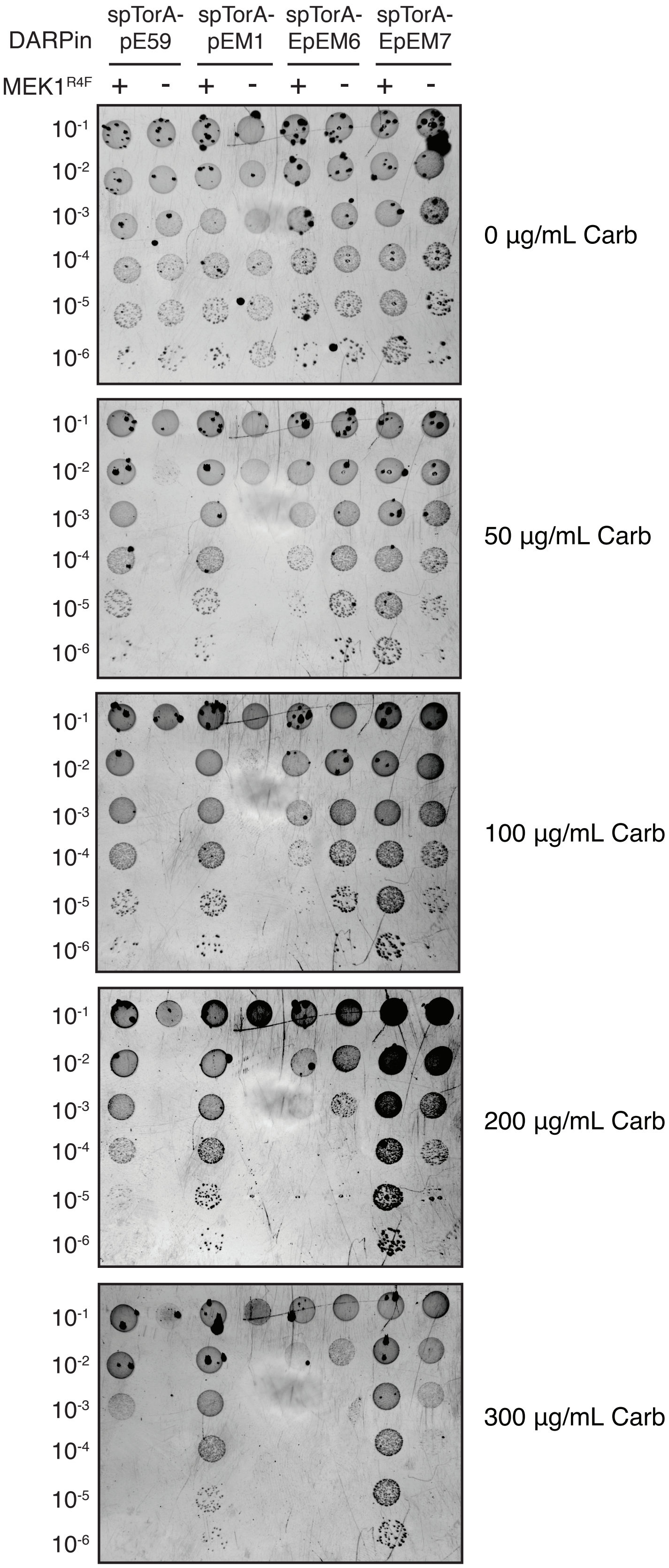

Supplementary Figure 6. Phenotypic selection of DARPin variants with altered specificity isolated using PhLI-TRAP. Representative spot titer images for serially diluted *E. coli* MC4100 cells co-expressing TatABC along with the antigen-Bla reporter fusion (ERK2-Bla) and a Tat-targeted DARPin (spTorA-pE59, spTorA-pEM1, spTorA-EpEM6, or spTorA-pEM7) as indicated. Resistance of cells was evaluated in the presence (+) or absence (-) of MEK1^{R4F} kinase. Overnight cultures were serially diluted in liquid LB and plated on LB agar supplemented with Carb (0-300 µg/ml).

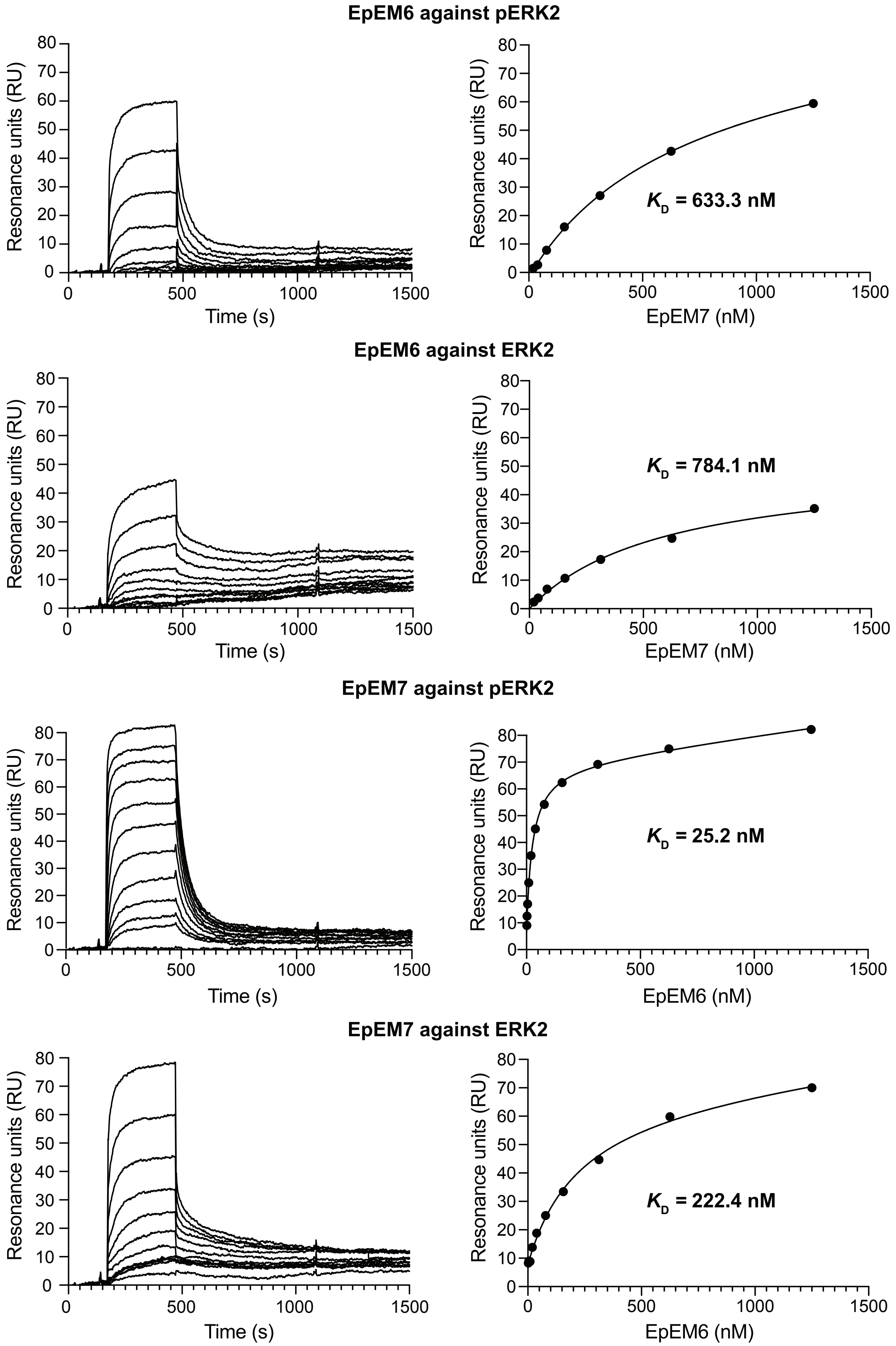

Supplementary Figure 7. Affinity determination of DARPin variants EpEM6 and EpEM7. The binding kinetics of variants EmEM6 and EpEM7 to the ERK2 and pERK2 antigens were monitored using Biacore. Biotinylated ERK2 and pERK2 were purified and immobilized at low concentrations, and the response of varied amounts of each DARPin (0, 1.2, 2.4, 4.9, 9.8, 19.5, 39, 78.125, 156.25, 312.5, 625, 1250 nM) was compared with an empty flow cell. Three independent experiments were carried out for each combination. Representative sensorgram results are depicted. The data were evaluated by fitting the equilibrium binding responses to obtain affinity values with resulting curve fits depicted to the right.

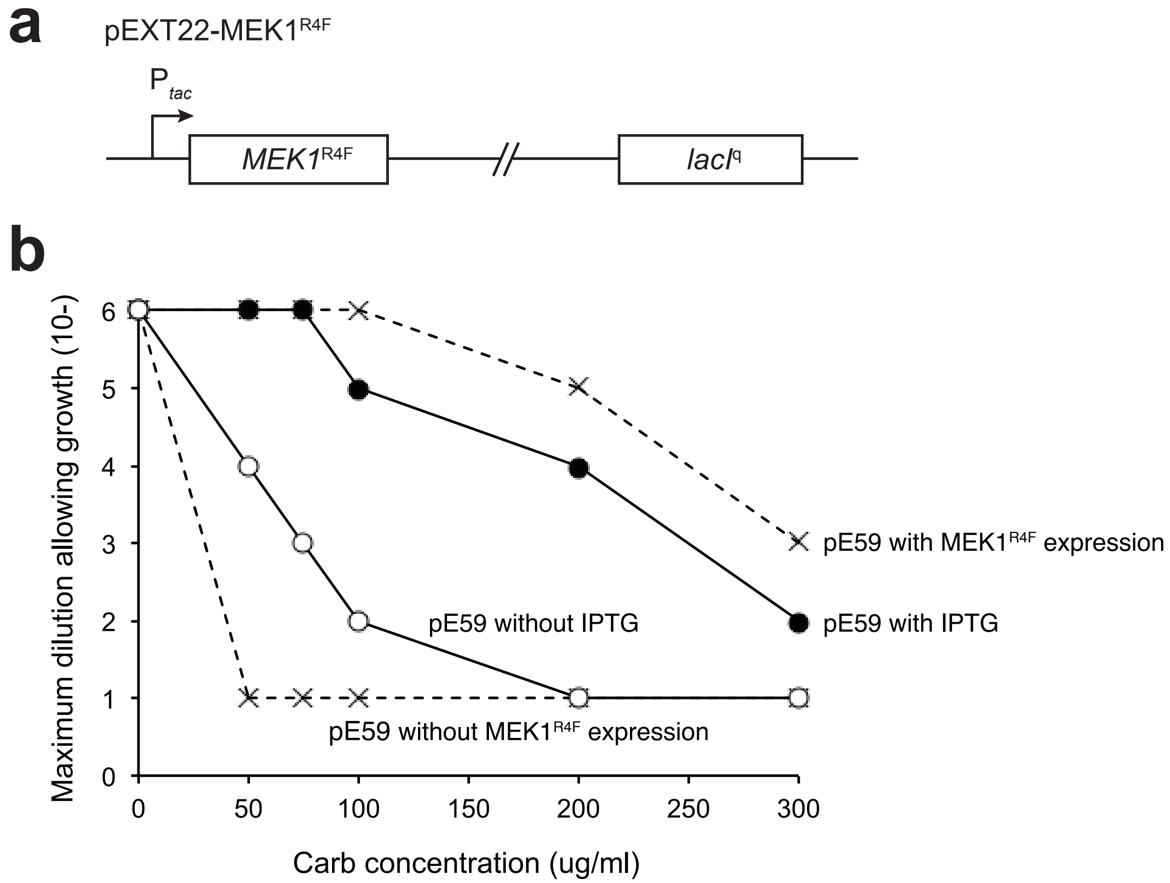

Supplementary Figure 8. Development of a selection/counterselection version of PhLI-TRAP strategy. (a) Schematic of plasmid pEXT22-MEK1^{R4F} that introduces P_{tac} promoter in front of MEK1^{R4F} gene for controlling expression of upstream kinase expression via addition of IPTG to cells. (b) Survival curves for serially diluted *E. coli* MC4100(DE3) cells co-expressing TatABC along with the antigen-Bla reporter fusion (ERK2-Bla) and Tat-targeted DARPin (spTorA-pE59) as indicated. Resistance of cells was evaluated in the presence (black circles) or absence (white circles) of IPTG. Survival curves for MC4100 cells carrying the original non-counterselectable PhLI-TRAP plasmids are shown for comparison (dashed lines with x marks). Maximal cell dilution that allowed growth is plotted versus Carb concentration.

Supplementary Table 1. Sequence analysis of selected DARPin clones

```
        N-capping repeat                Repeat 1
p59     DLGKKLLEAARAGQDDEVRILMANGADVNA  LDEDGLTPLHLAAQLGHLEIVEVLLKYGADVNA

pEM1    ------------------------------  ---------------------------------
pEM2    ------M-----------------------  -----------------------------G---
pEM3    ------------------------------  ------------------------V------K-

EpEM1   ---------------------I--------  ---------------------------------
EpEM2   ------------------------------  ---V-----------------------------
EpEM3   ----I-------------------------  ---V-----------------------------
EpEM4   ----------------------T-------  ---V-----------------------------
EpEM5   ---E-------------------D------  ---V-----------------------------
EpEM6   ------------------------------  ---V-----------------------------
EpEM7   -----P------------------------  -------------------------------Y-
        ***:  ***************:::******  *** *************************** *
EpE82   ------------------------------  F-QI-L-------FE-----------Y------
EpE89   ------------------------------  F-NI-L------SQW-----------H------

        Repeat 2                           C-capping repeat
pE59    EDNFGITPLHLAAIRGHLEIVEVLLKHGADVNA  QDKFGKTAFDISIDNGNEDLAEILQ

pEM1    ---Y-----------------------------  -------------------------
pEM2    ---------------------------------  -------------------------
pEM3    -------------------V-------------  -------------------------

EpEM1   ---Y-----------------------------  -------------------------
EpEM2   ---------------------------------  -------------------------
EpEM3   K--------------------------------  -------------------------
EpEM4   ---------------------------------  -------------------------
EpEM5   ---------------------------------  -------------------------
EpEM6   ---------------------------------  -----------------G-------
EpEM7   ---------------------------------  ----------------Y--------
        :**:*****************************  ****************  *******
EpE82   I-SY-I-------LH-----------Y------  -------------------------
EpE89   K-IY-I-------AK-----------H------  -------------------------
```

Supplementary Table 2. Primers used in this study

Primer Name	Sequence	Description
XbaI-RGS-6xHis-DARPin fwd	GCCATGTCTAGAAGAGGATCGCATCACCATC	To generate pDD18-spTorA-RGS-6xHis-DARPin or pDD18-spTorA-RGS-6xHis-DARPin::MEK1^{R4F}
SalI-stop-DARPin rev	GCCATGGTCGACTTAAAGCTTTTGCAGGATTTCAGCC	To generate pDD18-spTorA-RGS-6xHis-DARPin or pDD18-spTorA-RGS-6xHis-DARPin::MEK1^{R4F}
BamHI-OFF7 fwd	GCCATGGGATCCGATCTGGGACGTAAACTGCTG	To generate pDD18-spTorA-RGS-6xHis-OFF7 or pDD18-spTorA-RGS-6xHis-OFF7::MEK1^{R4F}
SalI-stop-OFF7 rev	GCCATGGTCGACGTTCAGTTTCTGCAGAATCTCTGC	To generate pDD18-spTorA-RGS-6xHis-OFF7 or pDD18-spTorA-RGS-6xHis-OFF7::MEK1^{R4F}
AvrII-ERK2-Bla fwd	GCCATGCCTAGGATGGCGGCGGCGG	To generate pDD322-TatABC::ERK2-Bla
XmaI-ERK2-Bla rev	GGCATGCCCGGGTTACCAATGCTTAATCAGTGAGGCAC	To generate pDD322-TatABC::ERK2-Bla
AvrII-MBP-Bla fwd	GCCATGCCTAGGATGAAAATCGAAGAAGGTAAACTGGTAATCTG	To generate pDD322-TatABC::MBP-Bla
XmaI-MBP-Bla rev	GGCATGCCCGGGTTACCAATGCTTAATCAGTGAGGCAC	To generate pDD322-TatABC::MBP-Bla
ERK2 T183E Y185F fwd primer	GATCATACAGGGTTCTTGGAAGAGTTTGTAGCCACGCGTTGG	To mutate phosphorylation sites at Thr183/Tyr185 to Glu183/Phe185 to generate pDD322-TatABC::mERK2-Bla
ERK2 T183E Y185F rev primer	CCAACGCGTGGCTACAAACTCTTCCAAGAACCCTGTATGATC	To mutate the phosphorylation sites at Thr183/Tyr185 to Glu183/Phe185 to generate pDD322-TatABC::mERK2-Bla
SbfI-RBS-XmaI-MEK1R4F fwd	GCCATGCCTGCAGGTTAAAGAGGAGAAAGGTCCCCGGGATGCCC AAGAAGAAGCCGACG	To generate pDD18-spTorA-RGS-6xHis-DARPin::MEK1^{R4F}
XmnI-HA tag-MEK1R4F rev	GCCATGGAAATATTTCTTAAGCGTAATCTGGAACATCGTATGGGTA GGCGGCGACGCCAGCAGCATGGG	To generate pDD18-spTorA-RGS-6xHis-DARPin::MEK1^{R4F}
XbaI-RGS-6xHis-pE59 fwd	GCCATGTCTAGAAGAGGATCGCATCACCATC	To perform error-prone PCR mutagenesis of pDD18-spTorA-RGS-6xHis-pE59 or pDD18-spTorA-RGS-6xHis-pE59::MEK1^{R4F}
SalI-stop-pE59	GCCATGGTCGACTTAAAGCTTTTGCAGGATTTCAGCC	To perform error-prone PCR mutagenesis of pDD18-spTorA-RGS-6xHis-pE59 or pDD18-spTorA-RGS-6xHis-pE59::MEK1^{R4F}
BamHI-pE59/pEM1/pEM2/pEM3 fwd	GCCATGGGATCCGACTTGGGTAAGAAACTGC	To generate pDST67-pE59/pEM1/pEM2/pEM3 for ELISA and SPR analysis
HindIII-pE59/pEM1/pEM2/pEM3 rev	GCCATGAAGCTTTTGCAGGATTTCAGCCAGG	To generate pDST67-pE59/pEM1/pEM2/pEM3 for ELISA and SPR analysis
BamHI-EpEM6 fwd	GCCATGGGATCCGACTTGGGTAAGAAACTG	To generate pDST67-EpEM6 for ELISA and SPR analysis
BamHI-EpEM7 fwd	GCCATGGGATCCGACTTGGGTAAGAAACCG	To generate pDST67-EpEM7 for ELISA and SPR analysis
HindIII-EpEM6/EpEM7 rev	GCCATGAAGCTTTTGCAGGATTTCAGC	To generate pDST67-EpEM6/EpEM7 for ELISA and SPR analysis
XbaI-MEK1R4F fwd	GCCATGTCTAGAATGCCCAAGAAGAAGCCGAC	To generate pEXT22-MEK1^{R4F} for IPTG inducible MEK1^{R4F} expression
HindIII–Stop-Myc tag-MEK1R4F	GCCATGAAGCTTTTACAGATCCTCTTCTGAGATGAGTTTTTGTTCA GCAGCGACGCCAGCAGCATGG	To generate pEXT22-MEK1^{R4F} for IPTG inducible MEK1^{R4F} expression
spTorA-RRtoKK fwd	CGATCTCTTTCAGGCATCAAAGAAGCGTTTTCTGGCACAACTCGG CGG	To generate export-defective signal peptide for plasmid spTorA(KK)-pE59
spTorA-RRtoKK rev	CCGCCGAGTTGTGCCAGAAAACGCTTCTTTGATGCCTGAAAGAGA TCG	To generate export-defective signal peptide for plasmid spTorA(KK)-pE59
