# Supplementary material for: A survival selection strategy for engineering synthetic binding proteins that specifically recognize post-translationally phosphorylated proteins
Source: Nat Commun. 2019 Apr 23;10:1830. doi: 10.1038/s41467-019-09854-y (PMC6478843; doi:10.1038/s41467-019-09854-y)
Supplement: Supplementary file 3 — Reporting Summary [file 41467_2019_9854_MOESM3_ESM.pdf]

nature research

Corresponding author(s): Matthew DeLisa
NCOMMS-18-27762A

Reporting Summary

Nature Research wishes to improve the reproducibility of the work that we publish. This form provides structure for consistency and transparency in reporting. For further information on Nature Research policies, see Authors & Referees and the Editorial Policy Checklist.

Statistical parameters

When statistical analyses are reported, confirm that the following items are present in the relevant location (e.g. figure legend, table legend, main text, or Methods section).

n/a	Confirmed	
☐	☒	The exact sample size (n) for each experimental group/condition, given as a discrete number and unit of measurement
☐	☒	An indication of whether measurements were taken from distinct samples or whether the same sample was measured repeatedly
☒	☐	The statistical test(s) used AND whether they are one- or two-sided<br>*Only common tests should be described solely by name; describe more complex techniques in the Methods section.*
☒	☐	A description of all covariates tested
☒	☐	A description of any assumptions or corrections, such as tests of normality and adjustment for multiple comparisons
☐	☒	A full description of the statistics including central tendency (e.g. means) or other basic estimates (e.g. regression coefficient) AND variation (e.g. standard deviation) or associated estimates of uncertainty (e.g. confidence intervals)
☒	☐	For null hypothesis testing, the test statistic (e.g. F, t, r) with confidence intervals, effect sizes, degrees of freedom and P value noted<br>*Give P values as exact values whenever suitable.*
☒	☐	For Bayesian analysis, information on the choice of priors and Markov chain Monte Carlo settings
☒	☐	For hierarchical and complex designs, identification of the appropriate level for tests and full reporting of outcomes
☒	☐	Estimates of effect sizes (e.g. Cohen's d, Pearson's r), indicating how they were calculated
☐	☒	Clearly defined error bars<br>*State explicitly what error bars represent (e.g. SD, SE, CI)*

Our web collection on statistics for biologists may be useful.

Software and code

Policy information about availability of computer code

Data collection	BIAevaluation software (GE Healthcare)
Data analysis	Prism 8.01 for macOS, Microsoft Excel for Mac version 16.21

For manuscripts utilizing custom algorithms or software that are central to the research but not yet described in published literature, software must be made available to editors/reviewers upon request. We strongly encourage code deposition in a community repository (e.g. GitHub). See the Nature Research guidelines for submitting code & software for further information.

Data

Policy information about availability of data

All manuscripts must include a data availability statement. This statement should provide the following information, where applicable:

- Accession codes, unique identifiers, or web links for publicly available datasets
- A list of figures that have associated raw data
- A description of any restrictions on data availability

All data generated or analyzed during this study are included in this article (and its supplementary information) or are available from the corresponding authors on reasonable request. The source data underlying Figs 2a, 2b, 3, 4a, 4b and 5a are provided as a Source Data file.

Field-specific reporting

Please select the best fit for your research. If you are not sure, read the appropriate sections before making your selection.

☒ Life sciences ☐ Behavioural & social sciences ☐ Ecological, evolutionary & environmental sciences

For a reference copy of the document with all sections, see nature.com/authors/policies/ReportingSummary-flat.pdf

Life sciences study design

All studies must disclose on these points even when the disclosure is negative.

Sample size	Minimally, sample sizes were chosen as three biological replicates; however, in some cases more than three biological replicates were performed. For the type of data presented here (replica plating of bacteria, Western blots, ELISA, and SPR), three replicates is the generally accepted norm in the field.
Data exclusions	No data was excluded in this work.
Replication	To verify the reproducibility of all results presented in the paper (replica plating of bacteria, Western blots, ELISA, and SPR), we performed three biological replicates of each. In every experiment presented, the results were found to be reproducible.
Randomization	Randomization is not a common practice for the type of molecular biological research described in this work and thus was not performed.
Blinding	Blinding is not a common practice for the type of molecular biological research described in this work and thus was not performed.

Reporting for specific materials, systems and methods

Materials & experimental systems

n/a	Involved in the study	
☒	☐	Unique biological materials
☐	☒	Antibodies
☒	☐	Eukaryotic cell lines
☒	☐	Palaeontology
☒	☐	Animals and other organisms
☒	☐	Human research participants

Methods

n/a	Involved in the study	
☒	☐	ChIP-seq
☒	☐	Flow cytometry
☒	☐	MRI-based neuroimaging

Antibodies

Antibodies used	Membranes were probed with the following antibodies: rabbit anti-p44/42 MAPK (Erk1/2) antibody (Cell Signaling; cat # 4695S) at 1/2,500 dilution to detect ERK2-Bla fusion; rabbit anti-p-p44/42 MAPK (Erk1/2) (Cell Signaling; cat # 9101S) at 1/2,500 dilution to detect pERK2-Bla fusion; mouse anti-RGS-4xHis (Qiagen; cat # 34610) at 1/2,500 dilution to detect DARPins; and rabbit anti-GroEL (Abcam; cat # ab90522) at 1/30,000 dilution to detect GroEL, which served as a fractionation marker.
Validation	The two antibodies from Cell Signaling are comprehensively validated for quality and performance (specificity,sensitivity, cross-reactivity) as discussed on their website (https://www.cellsignal.com/products/primary-antibodies/p44-42-mapk-erk1-2-137f5-rabbit-mab/4695) and (https://www.cellsignal.com/products/primary-antibodies/phospho-p44-42-mapk-erk1-2-thr202-tyr204-antibody/9101?site-search-type=Products&N=4294956287&Ntt=9101s&fromPage=plp&_requestid=3025773). Detailed protocols for usage can also be found at these websites. Likewise, the Qiagen and Abcam antibodies are comprehensively validated for quality and performance (specificity,sensitivity, cross-reactivity) as discussed on their websites (https://www.qiagen.com/us/shop/sample-technologies/protein/expression-purification-detection/rgs-his-antibody/#productdetails) and (https://www.abcam.com/groel-antibody-ab90522.html).
